# Re-evaluating haemolysis rejection criteria for PT, aPTT, and D-dimer testing: evidence from paired clinical samples on the CN-3000 analyser

**DOI:** 10.1515/almed-2026-0028

**Published:** 2026-05-14

**Authors:** Jirapa Kaewkhruawan, Wararat Masalae, Surapat Tanticharoenkarn, Pitchayaporn Riyagoon, Waroonkarn Laiklang, Kwanlada Chaiwong, Suppakorn Wongkamchai, Phandee Watanaboonyongcharoen, Chutitorn Ketloy, Orakan Limpornpugdee, Eakachai Prompetchara

**Affiliations:** Division of Laboratory Medicine, King Chulalongkorn Memorial Hospital, Bangkok, Thailand; Department of Laboratory Medicine, Faculty of Medicine, Chulalongkorn University, Bangkok, Thailand

**Keywords:** coagulation tests, haemolysis, preanalytical phase, sample rejection, analyser flagging

## Abstract

**Objectives:**

Haemolysis is the leading preanalytical cause of sample rejection in coagulation testing. This study prospectively evaluated the impact of spontaneous haemolysis on routine haemostasis assays by comparing paired haemolysed (H) and non-haemolysed (NH) clinical samples. Additionally, analyser flagging behaviour was characterised using an artificially haemolysed sample series.

**Methods:**

Thirty nine paired of H and NH samples were analysed for prothrombin time (PT), activated partial thromboplastin time (aPTT), and D-dimer (DD) on the Sysmex CN-3000. Mean biases were compared against predefined critical differences (CD) derived from biological variation data. Analyser flagging was additionally assessed using five sample pools across five haemolysis concentration levels (H1–H5).

**Results:**

When comparing H samples with NH samples, the mean bias was +0.07 s (+0.14 %) for PT (p=0.5979), +0.44 s (+0.66 %) for aPTT (p=0.8704), and +0.72 mg/L FEU (+10.63 %) for D-dimer (p=0.0199). D-dimer showed the highest relative bias but remained below the CD (64.7 %). Degree of haemolysis did not predict the magnitude of interference across the range of haemoglobin (Hb) concentrations studied. CN-3000 flagging grades were constant and correlated free Hb and irrespective of baseline sample PT/aPTT/D-dimer level.

**Conclusions:**

Mild to moderate spontaneous haemolysis does not produce clinically significant interference in PT, aPTT, or D-dimer. CN-3000 flagging behaviour is predictable and grade-dependent. Re-evaluation of automatic haemolysis rejection thresholds is warranted.

## Introduction

The preanalytical phase represents the most error-prone stage of the laboratory testing cycle, contributing up to 70 % of all diagnostic errors [1], 2]. Among preanalytical interferences, haemolysis, the pathological or iatrogenic disruption of erythrocyte membranes with release of intracellular constituents into the surrounding plasma, is universally recognised as the most frequent cause of sample rejection, with reported rates ranging from 3 to 23 % across clinical laboratories [3], 4].

In the coagulation laboratory, haemolysis poses distinct analytical challenges. Free haemoglobin and phospholipid-rich erythrocyte membrane fragments may interfere with optical clot-detection systems by altering turbidimetric signals, potentially producing spurious prolongations or shortenings of clot-based assay results [5], 6]. Additionally, ADP and red cell phospholipids released during haemolysis can theoretically modulate phospholipid-dependent coagulation reactions [5], 7].

Despite these recognised risks, the scientific evidence underpinning current haemolysis rejection thresholds is predominantly derived from studies employing artificially haemolysed specimens, typically prepared by mechanical disruption or freeze-thaw cycling of donor red cells added to normal pooled plasma [7], 8]. Such models may not truly reflect real-world interference because they generate elevated concentrations of intracellular analytes including lactic dehydrogenase, potassium, and haemoglobin, in proportions that may not replicate *in vivo* haemolysis [8]. Consequently, rejection criteria developed on this basis may be excessively stringent for the management of spontaneously haemolysed clinical samples.

The operational consequences of over-rejection are substantial. Each rejected sample necessitates repeat venepuncture, adding patient discomfort, delaying clinical decisions, and increasing laboratory workload [9]. Patients with difficult venous access including critically ill, paediatric, and oncological patients bear a disproportionate burden. Furthermore, repeat collection itself introduces risk of further haemolysis, perpetuating the problem.

Contemporary guidance from the Clinical and Laboratory Standards Institute (CLSI) and the European Federation of Clinical Chemistry and Laboratory Medicine (EFLM) advocates a risk-based approach to interference management, but specific haemolysis thresholds for coagulation assays remain poorly defined and inconsistently applied [10]. In parallel, modern automated coagulation analysers incorporate built-in interference detection algorithms that generate operator alerts based on measured haemolysis indices; however, the clinical specificity of these flags for predicting true analytical error has not been well characterised across sample types or flag severity levels.

We previously encountered this problem in the context of our autoverification programme, in which mild to moderate haemolysis systematically triggered blocking rules and necessitated manual review [11]. This resulted in inappropriate blocking of autoverification, which is, on clinical grounds, should have been automatically verified and released. The present study was therefore designed to address these gaps using two complementary approaches: (i) a prospective paired-sample analysis to quantify the impact of spontaneous *in vivo* haemolysis on PT, aPTT, and D-dimer in routine clinical specimens; and (ii) a systematic evaluation of CN-3000 analyser flagging behaviour across a graded series of artificially haemolysed samples. This evaluation incorporated both high- and low-concentration sample matrices to determine whether baseline analyte levels influence flag generation. The findings are intended to reduce unnecessary sample rejection while maintaining the accuracy and clinical validity of coagulation test results.

## Materials and methods

### Study design and clinical sample collection

A prospective, paired-sample study was conducted at the Division of Laboratory Medicine, King Chulalongkorn Memorial Hospital, Bangkok, Thailand, from July to December 2025. Institutional ethics approval was obtained prior to commencement (approval number 0606/2025), and all procedures were conducted in accordance with the Declaration of Helsinki. Informed consent was waived, as the study utilised residual (leftover) clinical specimens only. Samples were eligible for inclusion if: (i) the primary submitted specimen was visually identified as haemolysed by trained laboratory staff at the point of post-centrifugation inspection; (ii) the non-haemolysed recollected sample was received within 4 h of the original draw. Samples were excluded if the recollected sample was itself visually haemolysed. Blood was collected into sodium citrate (3.2 %) vacutainer tubes (Greiner Bio-One GmbH, Kremsmuenster, Austria) and processed within 2 h according to CLSI H21-A5 [12].

### Haemolysis assessment

Visual haemolysis assessment was performed by trained laboratory personnel after samples were centrifuged at 1,500×*g* for 15 min. The degree of plasma discoloration was evaluated semiquantitatively by comparison with a validated haemolysis reference color chart (Abbott Diagnostics, Wiesbaden, Germany), with minor internal modifications. This chart provides a standardized series of plasma color references corresponding to defined ranges of free haemoglobin (Hb) concentration.

According to our routine coagulation testing criteria, haemolysed samples were rejected when the plasma color reached ≥1+ on the haemolysis index (HI) scale indicated on the color chart, corresponding to an Hb concentration ≥0.3 g/L.

Following visual inspection, quantitative HI was measured using the Alinity c clinical chemistry analyser (Abbott Diagnostics, Wiesbaden, Germany) to confirm plasma Hb concentrations. A validated bichromatic spectrophotometric method was employed, measuring the differential absorbance of plasma at 570 and 600 nm, and converting the absorbance values into an estimated free haemoglobin concentration (g/L).

### Artificial haemolysis series for analyser flag characterisation

To characterise the flagging behaviour of the Sysmex CN-3000 system across defined haemolysis grades, an artificial haemolysis series was prepared. Five haemolysate concentrations (H1–H5) were generated using two freeze–thaw cycles of packed red blood cells, following the previously described protocol [13] with minor modifications. Briefly, approximately 5 mL of packed red blood cells from a donor were resuspended in physiological saline solution and centrifuged at 2,300×*g* for 5 min. The washing step was repeated three times. The washed red blood cells were then lysed by adding 10 mL of distilled water, mixed thoroughly, and stored at −20 °C for at least 12 h. After thawing, the sample was vigorously vortexed and subjected to a second freeze–thaw cycle (−20 °C for an additional 12 h). Following two freeze–thaw cycles, the lysed cells were centrifuged again at 1,500×*g* for 15 min to remove cellular debris. These steps yielded a stock haemolysate with a Hb concentration of 2.9 g/L.

The haemolysate was then 2-fold diluted with saline solution to generate the difference a different levels of haemolysis spanning high-grade (H1), intermediate (H2, H3) through low-grade or absent haemolysis (H4–H5). Each haemolysate was spiked into each plasma pool at a 1:6 (v/v) ratio (100 µL haemolysate + 600 µL plasma).

Five plasma pools with distinctly different baseline coagulation profiles were spiked with each haemolysate concentration following the procedure described above. Pool 1 represented the highest baseline PT, aPTT, and D-dimer values, whereas Pool 5 represented the lowest, enabling assessment of whether baseline analyte levels influenced flag generation. Following 1:6 (v/v) spiking with each haemolysate, the final Hb concentrations were 0.414, 0.210, 0.104, 0.052, and <0.050 g/L, respectively. The sample preparation matrix is provided in Supplementary Table S1. Each combination was analysed on the CN-3000 for PT, aPTT, and D-dimer. Flags generated by the CN-3000 interference detection algorithm were recorded for each run, using the instrument’s standard flag scoring: flag code 0 (Hb <0.087 g/L), code 1 (Hb 0.087 – <0.2 g/L), code 2 (Hb 0.2 – <0.312 g/L), code 3 (Hb 0.312 – <0.425 g/L), code 4 (Hb 0.425 – <0.538 g/L), and code 5 (Hb ≥0.538 g/L).

### Haemostasis assay methodology

All coagulation assays were performed on the Sysmex CN-3000 analyser (Sysmex Corporation, Kobe, Japan). PT was measured using Thromborel S, aPTT using Actin FS, and D-dimer using Innovance reagents. All reagents were from Siemens Healthineers, Marburg, Germany. Internal quality control was maintained per ISO 15189 requirements.

### Statistical analysis

Paired differences (H minus NH) were computed for each analyte. The Wilcoxon signed-rank test was applied with p<0.05 threshold for statistical significance. Relative biases were compared against critical differences (CD) derived from biological variation data [14]: PT 15.0 %, aPTT 10.0 %, D-dimer 64.7 %. Pearson correlation assessed the relationship between free Hb concentration and result bias. Analyses were performed using MedCalc Statistical Software v20 (MedCalc Software, Ostend, Belgium) and GraphPad Software Version 11.0.0 (La Jolla, CA, USA).

## Results

### Clinical sample characteristics

Thirty-nine paired H and NH sample sets were analysed. Median patient age was 58 years (IQR 42–71); 54 % were female. Most of the samples originated from outpatient department. Haemolysis flagged by CN-3000 analyser showed; 3 samples (7.7 %) code 1, 23 samples (59 %) code 2, 11 samples (28.2 %) code 3, and 2 samples (5.1 %) code 4.

### Bias analysis for haemostasis assays: clinical pairs

**Figure 1:**
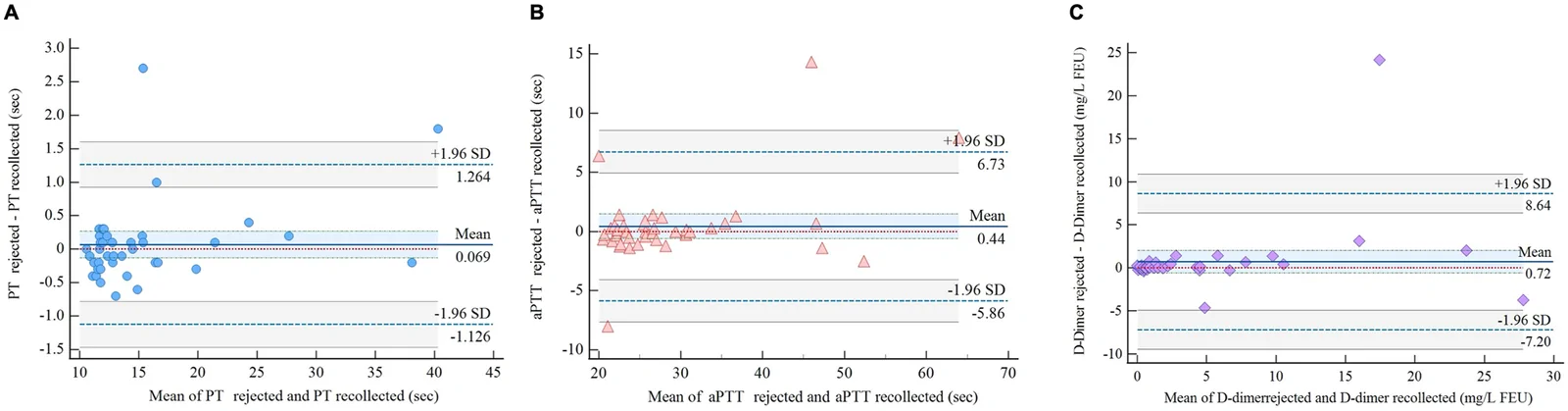
Bland-Altman analysis of agreement between haemolysed (rejected) and non-haemolysed (recollected) paired samples for routine haemostasis assays. Bland-Altman plots demonstrating the agreement between haemolysed (rejected) and non-haemolysed (recollected) paired samples for (A) PT, (B) aPTT, and (C) D-dimer (mg/L FEU). The x-axis represents the mean of each paired measurement, and the y-axis represents the difference (haemolysed minus non-haemolysed) for each pair (n=39). The solid blue line denotes the mean bias; the dashed red line and shaded blue region represent the limits of agreement (±1.96 SD) with their respective 95 % confidence intervals.

Table 1 summarises bias results. The mean PT bias was 0.07 s (0.14 %); 95 % CI: −0.13 to 0.27 s; p=0.5979, and the mean aPTT bias was 0.44 s (0.66 %); 95 % CI: −0.60 to 1.48; p=0.8704, both statistically and clinically non-significant. D-dimer showed a statistically significant mean bias of +0.72 mg/L FEU (10.63 %); 95 % CI: −0.59 to 2.03 mg/L; p=0.0199, see also Figure 1A–C, but the relative bias of 10.63 % remained well below the CD of 64.7 %. Importantly, although the bias of D-dimer reached statistical significance, its magnitude is not clinically meaningful, as it does not approach thresholds that would alter clinical interpretation or decision-making. In particular, such a degree of variation is unlikely to affect classification around commonly used diagnostic cut-offs or influence patient management. Therefore, the impact of haemolysis on D-dimer measurement in this context can be considered clinically negligible. Notably, two PT, one aPTT, and one D-dimer paired samples fell outside the limits of agreement (+1.96 SD) in the Bland–Altman analysis. Nevertheless, these differences did not alter the clinical interpretation of the test results.

**Table 1:** Mean bias, relative bias, and critical difference comparison for paired haemolysed and non-haemolysed samples.

Parameter	Mean bias (H vs. NH)	Relative bias, %	Critical difference, CD	p-Value
PT, s	0.07 s	0.14 %	15.0 %	0.5979
aPTT, s	0.44 s	0.66 %	10.0 %	0.8704
D-dimer, mg/L FEU	0.72 mg/L	10.63 %	64.7 %	0.0199^a^

^a^Statistically significant (p<0.05). H, haemolysed; NH, non-haemolysed; CD, critical difference from biological variation data [14]. FEU, fibrinogen equivalent units.

**Figure 2:**
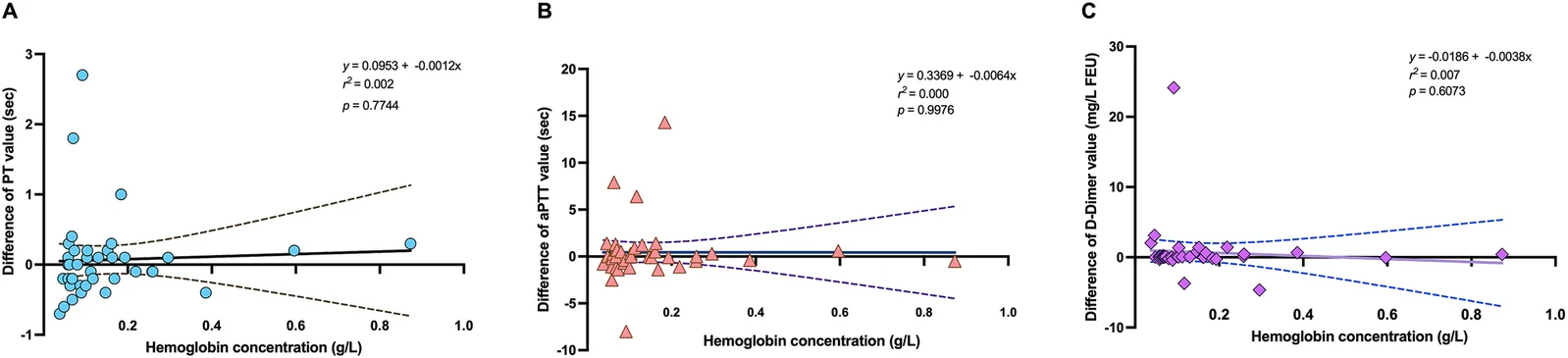
Correlation between plasma free Hb concentration and the difference in haemostasis assay results between haemolysed and non-haemolysed paired samples. Scatter plots illustrating the relationship between plasma free Hb concentration (g/L) and the paired difference (haemolysed minus non-haemolysed) for (A) PT, (B) aPTT, and (C) D-dimer (mg/L). Each data point represents one paired sample set (n=39). The solid black/blue line represents the linear regression line and dashed lines denote the 95 % confidence interval. Regression equations and coefficients of determination (r²) are displayed within each panel. No statistically significant correlation was observed between Hb concentration and the magnitude of assay difference.

No significant correlation was observed between free Hb concentration and bias magnitude for any analyte (PT: r^2^=0.002, p=0.7744; aPTT: r^2^=0.000, p=0.9976; D-dimer: r^2^=0.007, p=0.6073). This indicating that the degree of haemolysis did not predict the magnitude of interference across the range of haemoglobin concentrations studied. Correlation analysis between Hb concentration and paired differences is shown in Figure 2A–C.

### PT, aPTT, and D-dimer results in artificially haemolysed sample series

**Figure 3:**
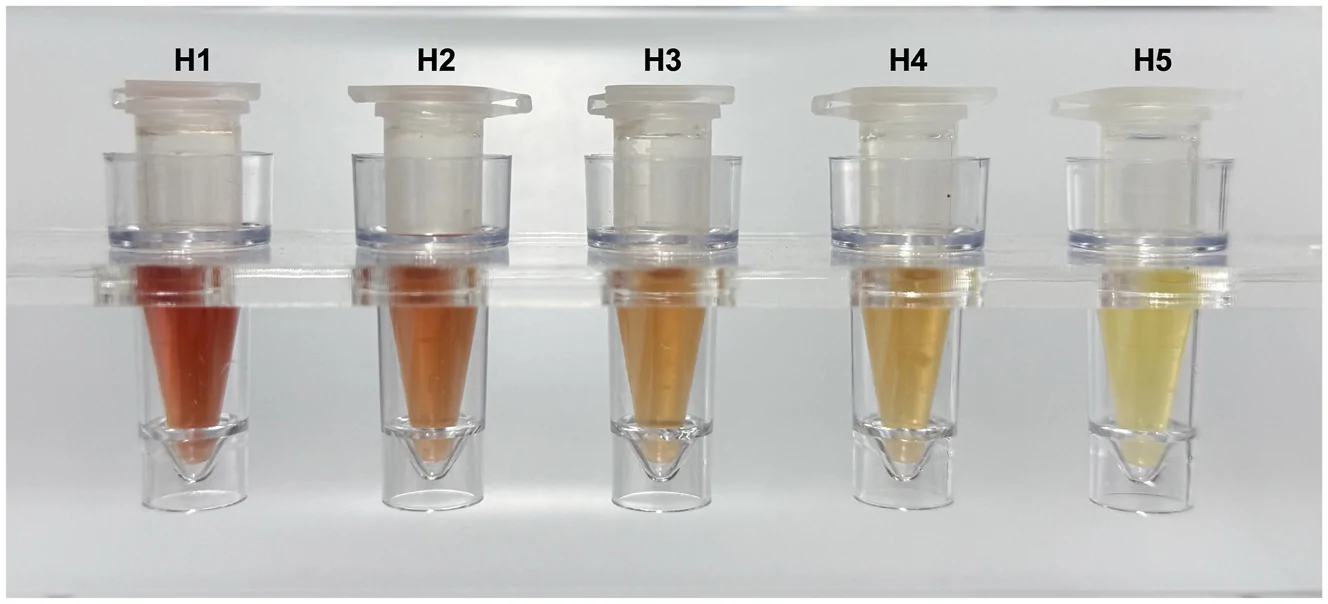
Visual appearance of artificially haemolysed plasma samples across five haemolysis grades (H1–H5). Five haemolysis levels (H1–H5) were generated using serial dilutions of an artificially prepared haemolysate. The plasma samples showed visually distinguishable colour intensities corresponding to different haemoglobin (Hb) concentrations: H1=0.414 g/L, H2=0.210 g/L, H3=0.104 g/L, H4=0.052 g/L, and H5<0.050 g/L. This graded haemolysis series was used to systematically evaluate the flagging behaviour of the CN-3000 analyser across increasing levels of haemolysis severity.

Table 2 presents PT, aPTT, and D-dimer values across the five haemolysate concentrations (H1–H5) (Figure 3) and five sample pools (Pool 1–Pool 5). Across all three analytes, PT, aPTT, and D-dimer (DD) results showed minimal and non-directional variation across haemolysis concentrations within each sample pool. For example, PT results for Pool 1 ranged from 21.4 to 22.0 s across H1–H5, and for Pool 5 from 12.5 to 12.7 s. Similarly, aPTT values for Pool 1 ranged 36.3–37.8 s, and D-dimer values for Pool 1 ranged 3.08–3.27 mg/L FEU. No systematic trend of progressive result elevation or reduction with increasing haemolysate concentration was apparent, consistent with the absence of dose-dependent interference within this haemolysis range.

**Table 2:** Effect of graded haemolysis on PT, aPTT, and D-dimer across plasma pools.

Sample pool	Hb, g/L	PT, s	aPTT, s	D-dimer, mg/L FEU
Pool 1	H1 (0.429)	21.7	37.8	3.21
	H2 (0.214)	22	37.8	3.21
	H3 (0.107)	21.6	36.3	3.24
	H4 (0.054)	21.7	37	3.08
	H5 (<0.05)	21.4	36.5	3.27
Pool 2	H1 (0.429)	16.9	31.9	2.94
	H2 (0.214)	17.4	32.8	3.02
	H3 (0.107)	17.1	31.1	3.02
	H4 (0.054)	17	31	3.17
	H5 (<0.05)	17	32	2.8
Pool 3	H1 (0.429)	14.6	28.5	2.8
	H2 (0.214)	14.5	27.7	2.54
	H3 (0.107)	14.3	27.1	2.67
	H4 (0.054)	14.6	28.6	2.64
	H5 (<0.05)	14.4	27.5	2.5
Pool 4	H1 (0.429)	13.3	26.2	2.6
	H2 (0.214)	13.3	25.5	2.43
	H3 (0.107)	13.3	25.6	2.47
	H4 (0.054)	13.2	25.1	2.45
	H5 (<0.05)	13.1	25.1	2.47
Pool 5	H1 (0.429)	12.7	23.8	2.45
	H2 (0.214)	12.6	23.7	2.36
	H3 (0.107)	12.5	23.7	2.36
	H4 (0.054)	12.7	23.7	2.4
	H5 (<0.05)	12.7	23.7	2.45

H1–H5, final Hb concentrations from highest (H1, 0.414 g/L; grade 3) to lowest (H5, <0.05 g/L; no flag).

### CN-3000 analyser flagging according to haemolysis grade

Table 3 presents CN-3000 flagging results for the artificially haemolysed series. The flagging pattern was identical across all five sample pools (Pool 1–5) and for both ‘High’ and ‘Low’ PT/aPTT/D-dimer value categories, indicating that baseline analyte level does not modulate haemolysis flag generation on the analyser.

**Table 3:** CN-3000 analyser flag codes for artificially haemolysed samples across haemolysis concentrations (H1–H5) and sample pools (Pool 1–5).

Sample	H1 (Hb 0.414 g/L)	H2 (Hb 0.210 g/L)	H3 (Hb 0.104 g/L)	H4 (Hb 0.052 g/L)	H5 (Hb <0.05 g/L)
	←High Hb	Intermediate	Low Hb	Low Hb	Low Hb→
Pool 1	3	2	1	No flag	No flag
Pool 2	3	2	1	No flag	No flag
Pool 3	3	2	1	No flag	No flag
Pool 4	3	2	1	No flag	No flag
Pool 5	3	2	1	No flag	No flag

Flag codes: 3, high-Hb interference alert; 2 and 1, moderate to mild haemolysis warning. Flags were identical for all five sample pools and both high and low PT/aPTT/DD value categories, indicating flag generation is independent of baseline analyte level. Hb, haemoglobin.

At H1 (highest free Hb, 0.410 g/L), flag code 3 was generated for all five sample pools across all three analytes representing high-confidence interference alert. At H2 and H3, flags code 2 and 1 were triggered, respectively, indicating a moderate/mild haemolysis warning. At H4 and H5, no flags were generated. This graded, reproducible flagging pattern demonstrates that the CN-3000 interference detection algorithm reliably escalates from no alert through mild and critical flag as haemolysis severity increases, independent of sample clinical context.

Importantly, cross-referencing the flagging data with the bias analysis from clinical paired samples reveals a key discordance, the CN-3000 generates flag code 1 to code 3 alerts, yet our clinical data demonstrate that at these haemolysis levels, no clinically significant bias in PT, aPTT, or D-dimer is present relative to the paired NH sample.

## Discussion

This study provides two complementary and clinically actionable datasets. First, prospective paired-sample analysis demonstrates that spontaneous haemolysis of mild to moderate severity produces no clinically meaningful bias in PT, aPTT, or D-dimer measurements on the Sysmex CN-3000 analyser. Second, the study systematically characterises the analyser’s haemolysis flagging behaviour, showing that interference flags are generated in a predictable, grade-dependent pattern that is independent of baseline analyte concentrations. Importantly, flag codes 1–3 observed in this study were not associated with clinically significant alterations in coagulation results.

The absence of clinically significant bias in the clinical paired analysis is consistent with prior literature using spontaneously haemolysed specimens [15], 16]. Previous investigations reported that clinically significant effects on PT and aPTT occur only when free haemoglobin exceeds approximately 5 g/L and 1.5 g/L, respectively [17]. In contrast, another large paired-sample study (n=296) performed on the ACL TOP 750-CTS demonstrated significant differences in aPTT and D-dimer values, while PT appeared less affected by haemolysis [18]. These discrepancies may partly reflect differences in reagent composition and analytical platforms, particularly the use of Synthasil reagent on the ACL TOP system compared with Actin FS reagent on the CN-3000 analyser. Variations in phospholipid composition and reagent sensitivity may influence the degree to which haemolysis affects clotting assays.

Our findings are consistent with previous studies using the Sysmex CN-6000 analyser, which reported minimal analytical impact of haemolysis on coagulation parameters. Specifically, PT, aPTT, and D-dimer values demonstrated less than 1 % bias when free haemoglobin concentrations were below 1 g/L [19]. In the present study, the highest free haemoglobin concentration observed in clinical samples was 0.87 g/L, which falls well below these previously reported interference thresholds. Therefore, samples within this haemolysis range are unlikely to produce clinically meaningful interference and should not necessarily be rejected.

The statistically significant increase observed in D-dimer values (+0.72 mg/L FEU; p=0.0199) warrants careful interpretation. Although statistically significant, the relative bias of 10.63 % remains substantially below the predefined critical difference (CD) of 64.7 % [14], indicating that the magnitude of change is unlikely to affect clinical interpretation in most cases. In clinical practice, D-dimer results are often interpreted within decision-making frameworks such as age-adjusted thresholds for venous thromboembolism exclusion. Within such contexts, a 10 % analytical bias would rarely alter clinical decision-making, except possibly in borderline cases near the diagnostic threshold [20]. The statistical significance without clinical significance observed here illustrates the essential distinction between analytical and clinical impact, a principle central to International Federation of Clinical Chemistry and Laboratory Medicine (IFCC) guidance on allowable error [21], [22], [23].

The artificial haemolysis experiments provide additional practical insights for laboratory operation. The finding that identical flag codes were generated across sample pools with markedly different baseline PT, aPTT, and D-dimer values indicates that the CN-3000 haemolysis detection algorithm functions independently of analyte concentration and relies primarily on spectrophotometric haemolysis index measurement. This observation has important practical implications: laboratories can apply a uniform interpretation strategy for haemolysis flags regardless of the underlying coagulation values or clinical indication for testing. Based on these data, mild to moderate haemolysis does not significantly alter test results and could therefore be incorporated into autoverification rules [11], potentially reducing unnecessary sample rejection, shortening turnaround time, and decreasing laboratory workload.

The present findings have direct implications for optimizing haemolysis management in routine coagulation testing across different clinical settings. Within the haemolysis range evaluated (free haemoglobin ≤0.87 g/L in clinical samples), no clinically meaningful bias was observed for PT, aPTT, or D-dimer, and lower-level haemolysis flags (codes 1–3) were not associated with significant analytical interference.

Accordingly, laboratories using the Sysmex CN-3000 or comparable optical detection systems may consider adopting a risk-stratified, flag-guided acceptance approach rather than blanket rejection. Specifically, samples with low to moderate haemolysis (e.g., HI corresponding to Hb <∼1.0 g/L or flag codes 1–3) may be accepted for analysis and autoverification, provided that results are interpreted in the appropriate clinical context. In contrast, samples exceeding this range (e.g., Hb ≥1.0 g/L or higher-grade flags) should be subject to manual review or repeat sampling, particularly when results are near clinical decision thresholds.

However, several limitations should be acknowledged. First, the relatively small number of paired clinical samples (n=39) limits statistical power, particularly for subgroup analyses, and larger multicentre studies are needed to validate and refine haemolysis-specific acceptance thresholds. Second, the study was conducted on a single analyser platform; therefore, the findings are platform-specific and should not be directly extrapolated to other systems, particularly those using mechanical clot detection or different haemolysis detection algorithms. In addition, the range of haemolysis evaluated was limited. The baseline coagulation values and haemolysis indices in this study did not include extremely elevated PT, aPTT, or D-dimer levels, nor very high haemolysis indices. The maximum free haemoglobin concentrations observed were 0.87 g/L in clinical samples and 0.410 g/L in the artificially haemolysed series. As such, the effects of more severe haemolysis remain uncertain and warrant further investigation. Furthermore, patients receiving direct oral anticoagulants (DOACs) were not specifically assessed in this study, and haemolysis-related interference patterns may differ in this population.

Taken together, these considerations indicate that the findings should be interpreted within the analytical and haemolysis ranges evaluated in this study. Within the haemolysis range commonly encountered in our laboratory, the results support the use of laboratory-specific evidence to refine haemolysis rejection criteria and optimise autoverification rules. Such adjustments may reduce unnecessary specimen rejection and manual review while maintaining the reliability of reported coagulation results. However, broader generalisation will require confirmation in larger, multi-platform studies encompassing a wider spectrum of haemolysis severity.

## Conclusions

This study demonstrates that mild to moderate spontaneous haemolysis does not produce clinically meaningful interference in PT, aPTT, or D-dimer measurements on the Sysmex CN-3000 analyser. Haemolysis flagging follows a reproducible, grade-dependent pattern independent of baseline analyte levels, and lower-level flags (codes 1–3) were not associated with clinically significant bias. These findings support reconsideration of haemolysis rejection policies and the potential implementation of flag-guided, risk-based acceptance strategies. Further multicentre and multi-platform studies are required to confirm generalisability and inform evidence-based guidelines.

## Supplementary Material

Supplementary Material
