# Supplementary material for: Re-evaluating haemolysis rejection criteria for PT, aPTT, and D-dimer testing: evidence from paired clinical samples on the CN-3000 analyser
Source: Adv Lab Med. 2026 May 14;7(3):179–86. doi: 10.1515/almed-2026-0028 (PMC13552810; doi:10.1515/almed-2026-0028)
Supplement: Supplementary file 1 [file j_almed-2026-0028_suppl_001.docx]

**Table S1:** Sample preparation matrix showing the generation of plasma pools with different baseline coagulation profiles and haemoglobin concentrations following haemolysate spiking.

|  | **Pool 1** | **Pool 2** | **Pool 3** | **Pool 4** | **Pool 5** |
| --- | --- | --- | --- | --- | --- |
| **Hemolysate 1**  (Hb 2.9 g/L) | **H1 100 µL + P1 600 µL**  (Hb 0.414 g/L) | **H1 100 µL + P2 600 µL**  (Hb 0.414 g/L) | **H1 100 µL + P3 600 µL**  (Hb 0.414 g/L) | **H1 100 µL + P4 600 µL**  (Hb 0.414 g/L) | **H1 100 µL + P5 600 µL**  (Hb 0.414 g/L) |
| **Hemolysate 2**  Hb 1.45 g/L | **H2 100 µL + P1 600 µL**  (Hb 0.210 g/L) | **H2 100 µL + P2 600 µL**  (Hb 0.210 g/L) | **H2 100 µL + P3 600 µL**  (Hb 0.210 g/L) | **H2 100 µL + P4 600 µL**  (Hb 0.210 g/L) | **H2 100 µL + P5 600 µL**  (Hb 0.210 g/L) |
| **Hemolysate 3**  Hb 0.725 g/L | **H3 100 µL + P1 600 µL**  (Hb 0.104 g/L) | **H3 100 µL + P2 600 µL**  (Hb 0.104 g/L) | **H3 100 µL + P3 600 µL**  (Hb 0.104 g/L) | **H3 100 µL + P4 600 µL**  (Hb 0.104 g/L) | **H3 100 µL + P5 600 µL**  (Hb 0.104 g/L) |
| **Hemolysate 4**  Hb 0.363 g/L | **H4 100 µL + P1 600 µL**  (Hb 0.052 g/L) | **H4 100 µL + P2 600 µL**  (Hb 0.052 g/L) | **H4 100 µL + P3 600 µL**  (Hb 0.052 g/L) | **H4 100 µL + P4 600 µL**  (Hb 0.052 g/L) | **H4 100 µL + P5 600 µL**  (Hb 0.052 g/L) |
| **Hemolysate 5**  Hb 0.181 g/L | **H5 100 µL + P1 600 µL**  (Hb <0.050g/L)) | **H5 100 µL + P2 600 µL**  (Hb <0.050g/L)) | **H5 100 µL + P3 600 µL**  (Hb <0.050g/L)) | **H5 100 µL + P4 600 µL**  (Hb <0.050g/L)) | **H5 100 µL + P5 600 µL**  (Hb <0.050g/L) |

Abbreviations: H; Hemolysate, P; Pool plasma. Pool 1 represented the highest baseline PT/aPTT/D-dimer values, and Pool 5 the lowest.

The final concentration of free Hb was indicated in parentheses.
